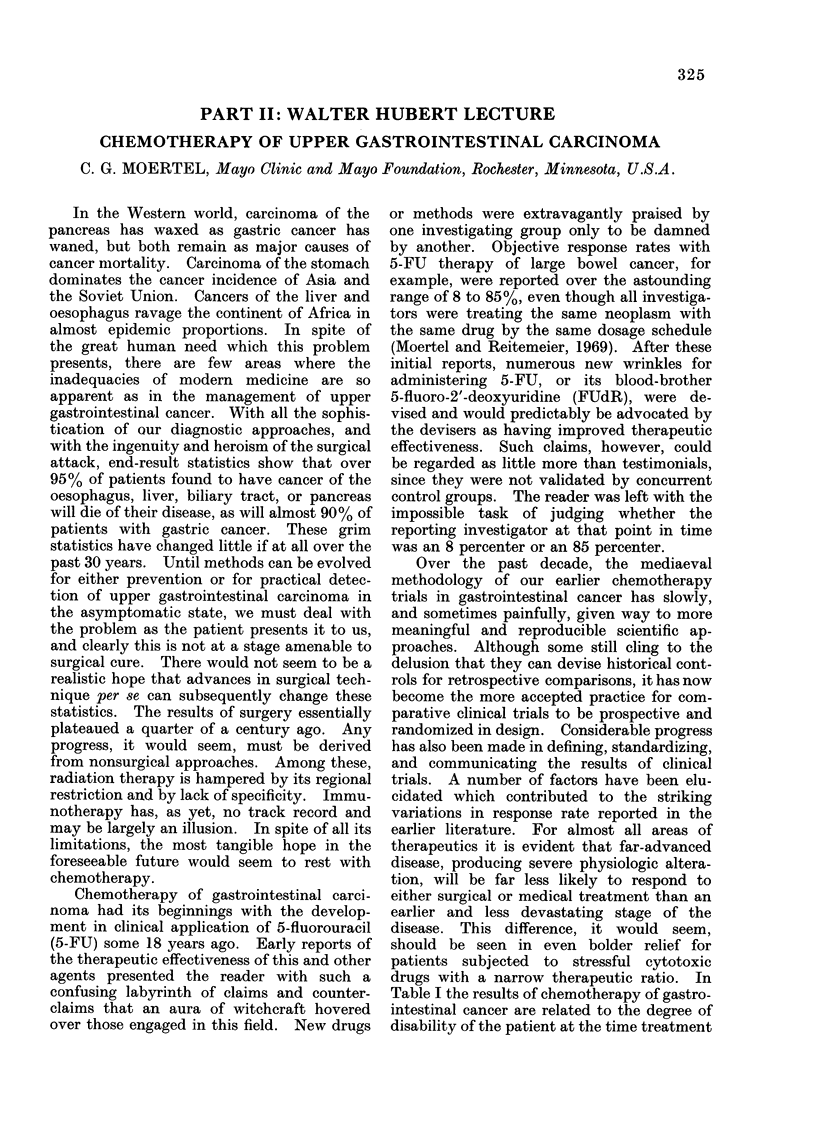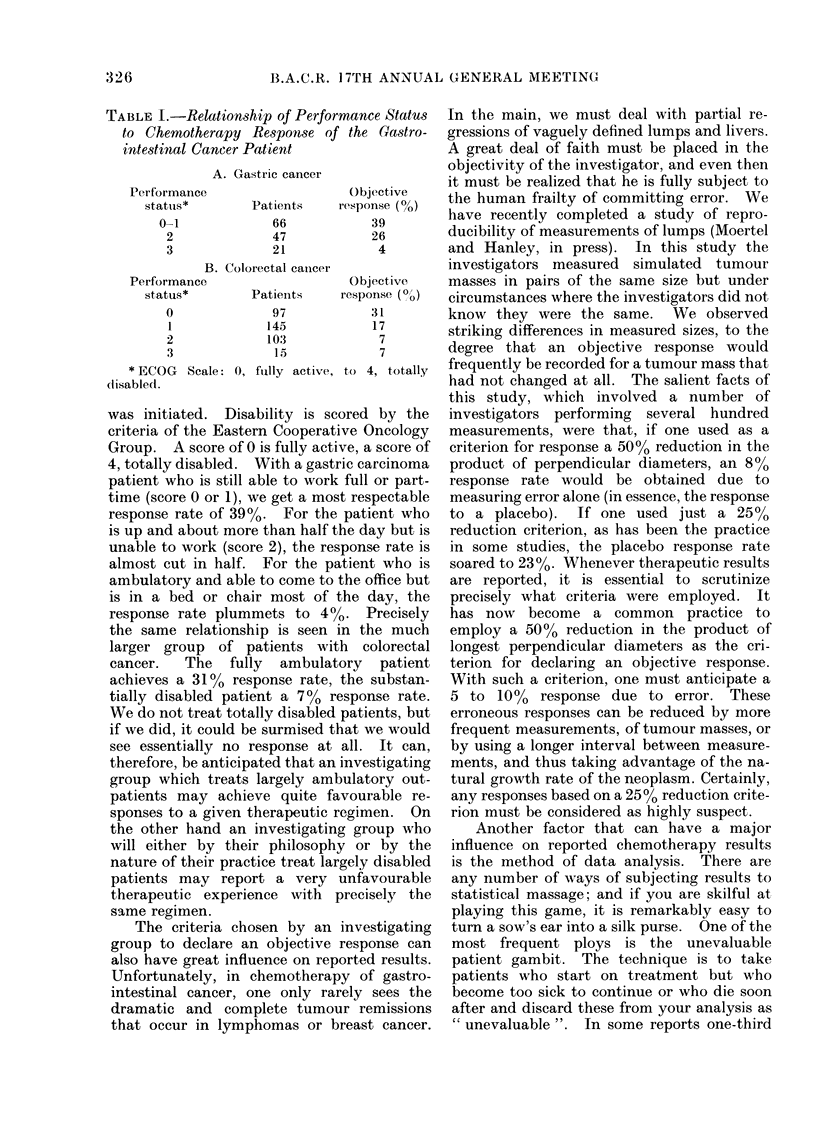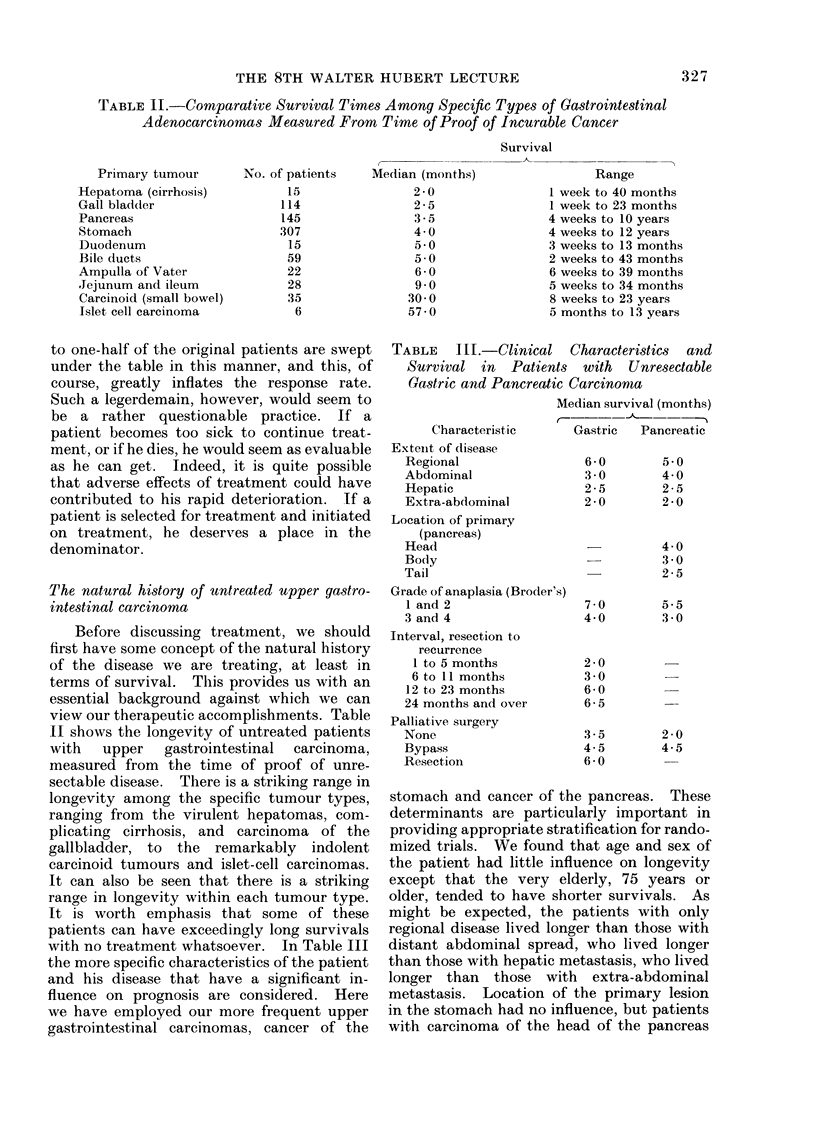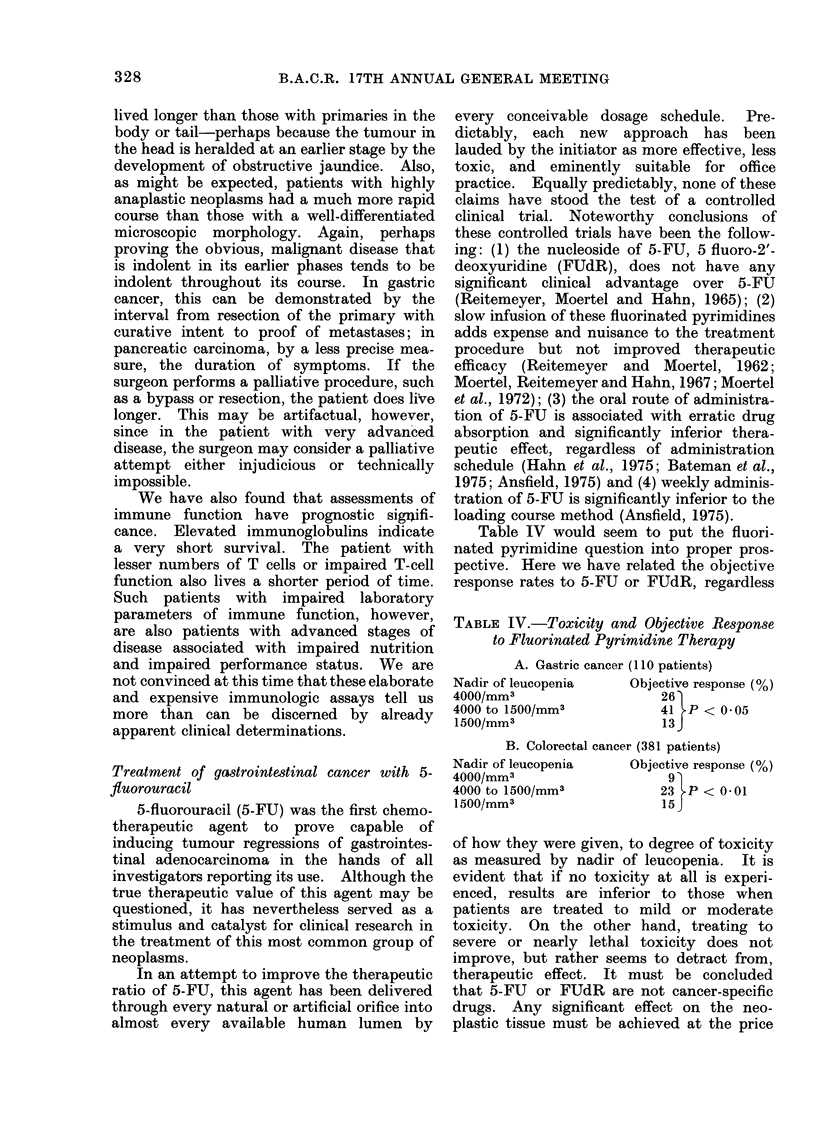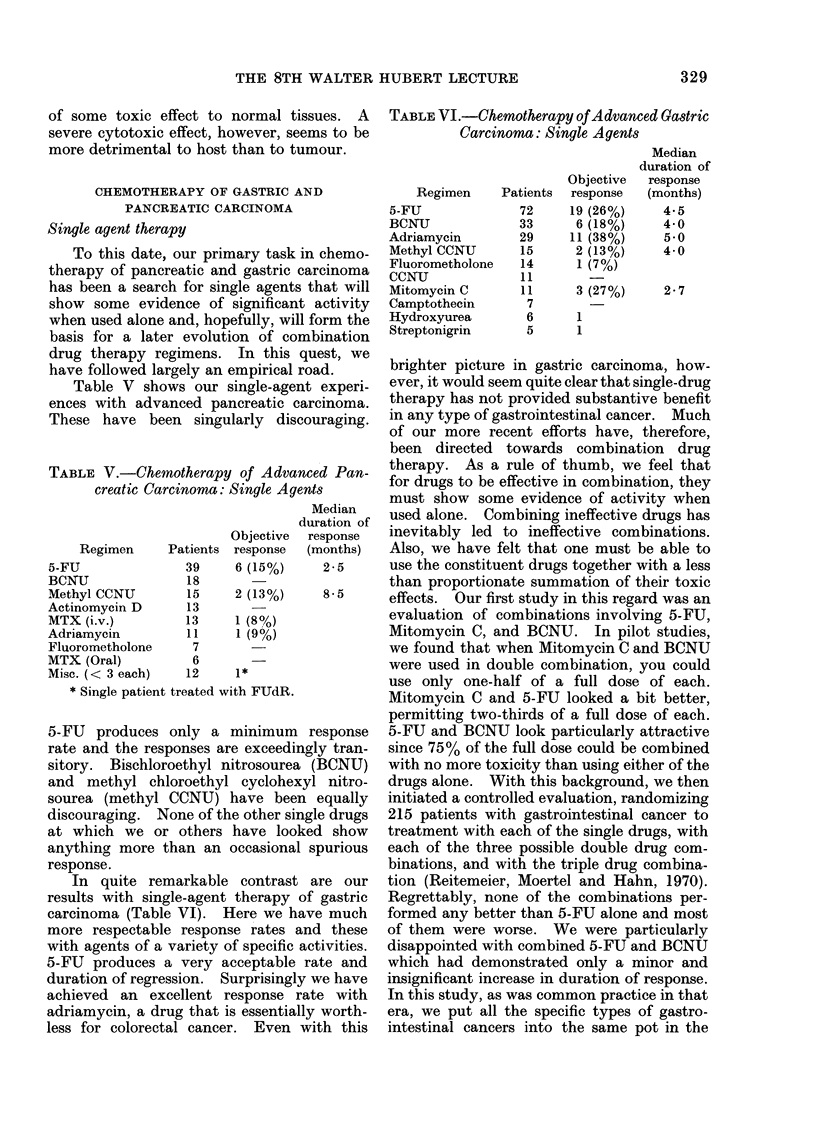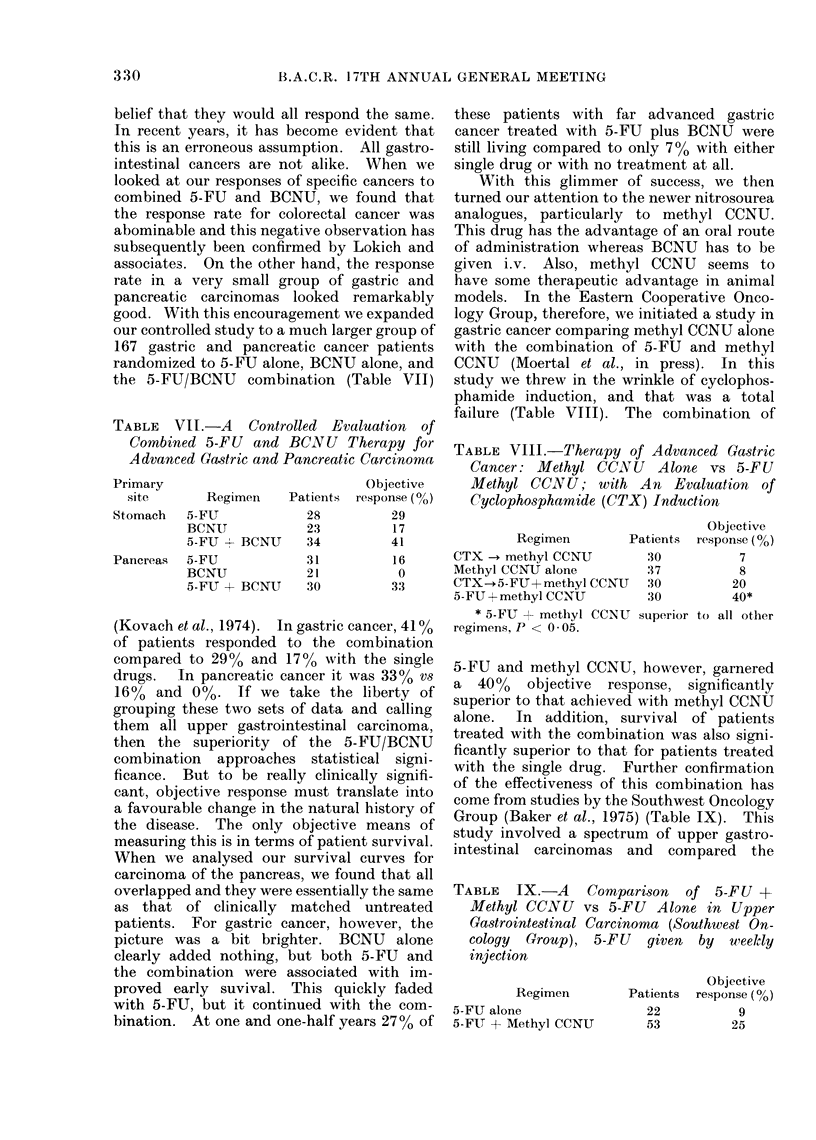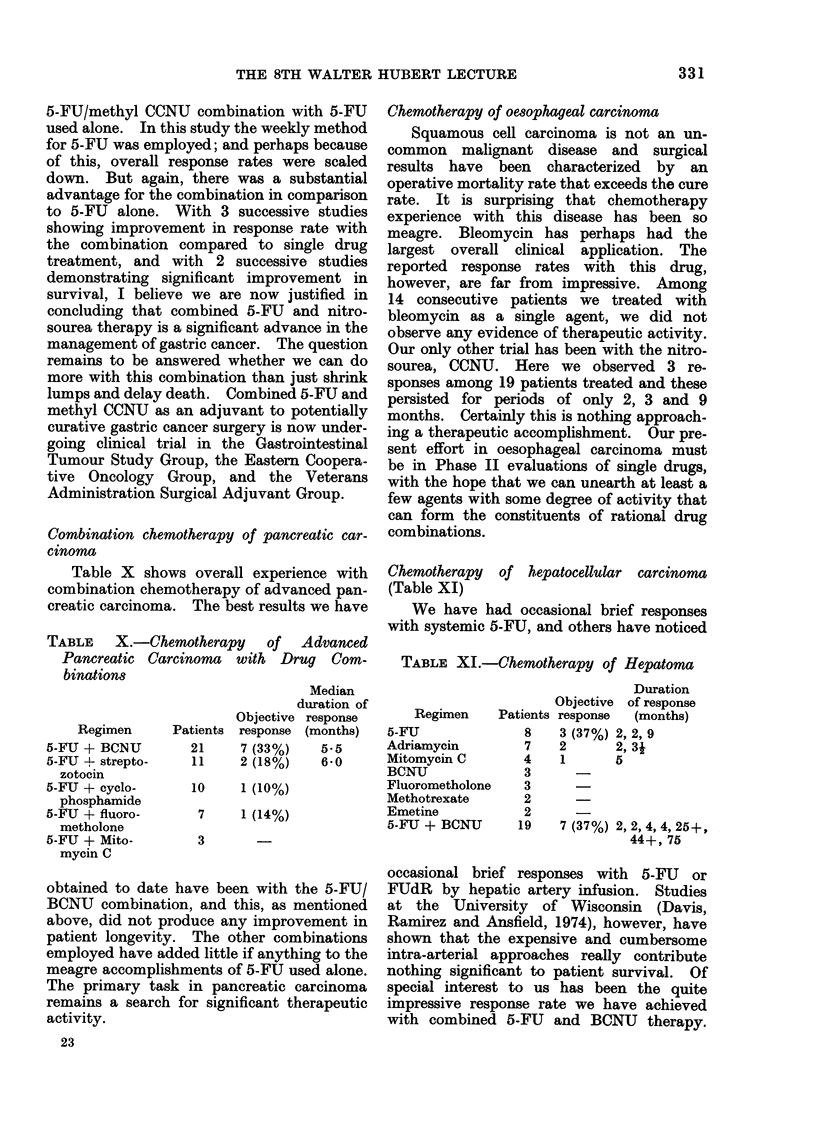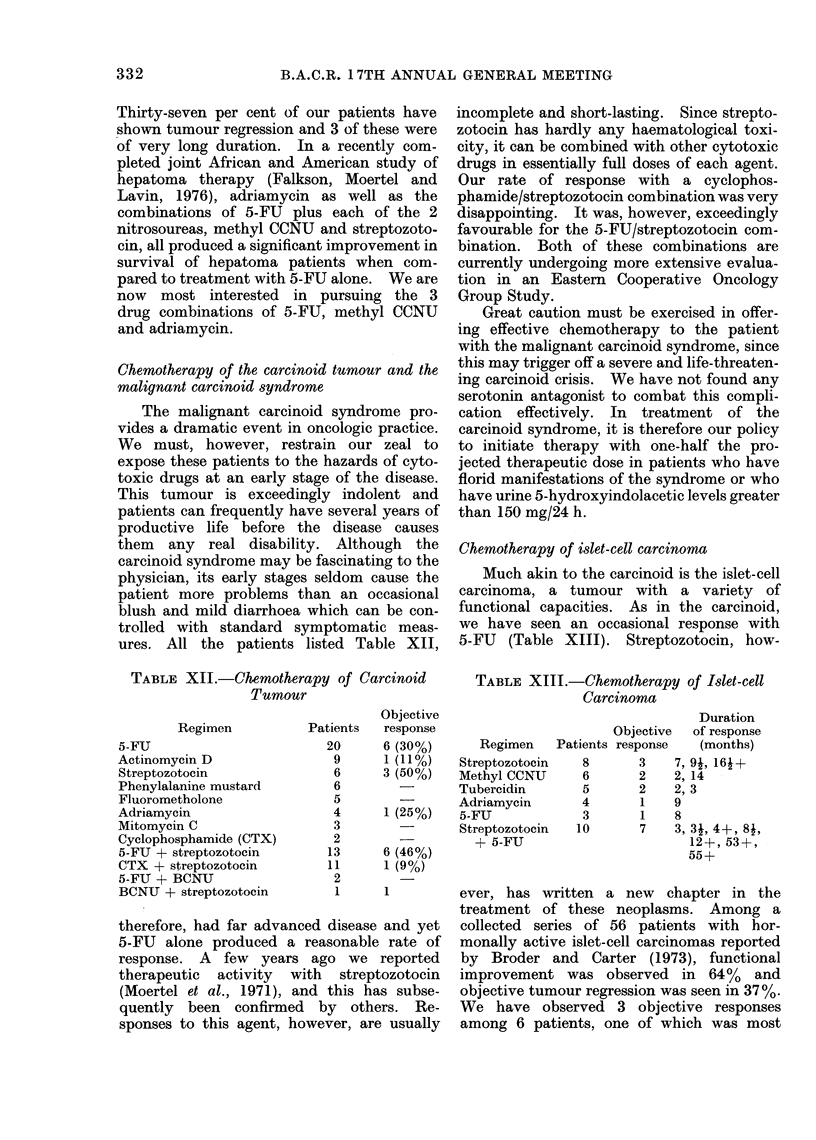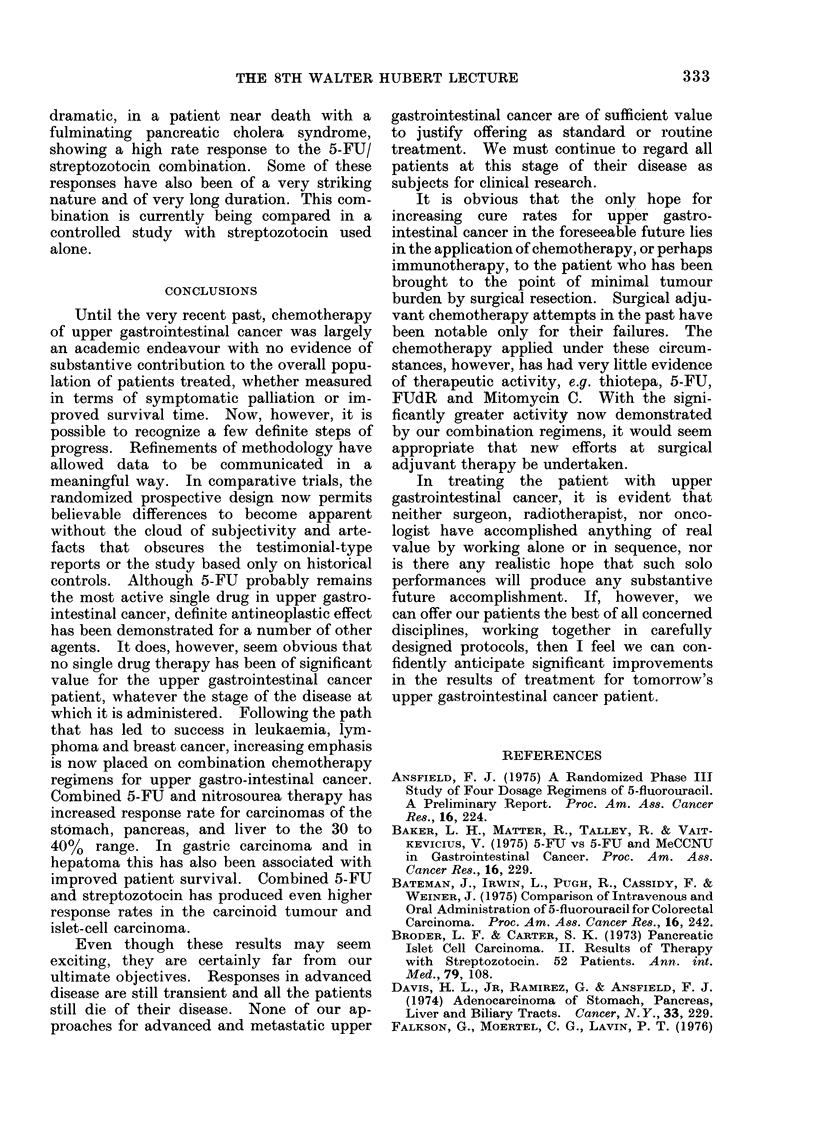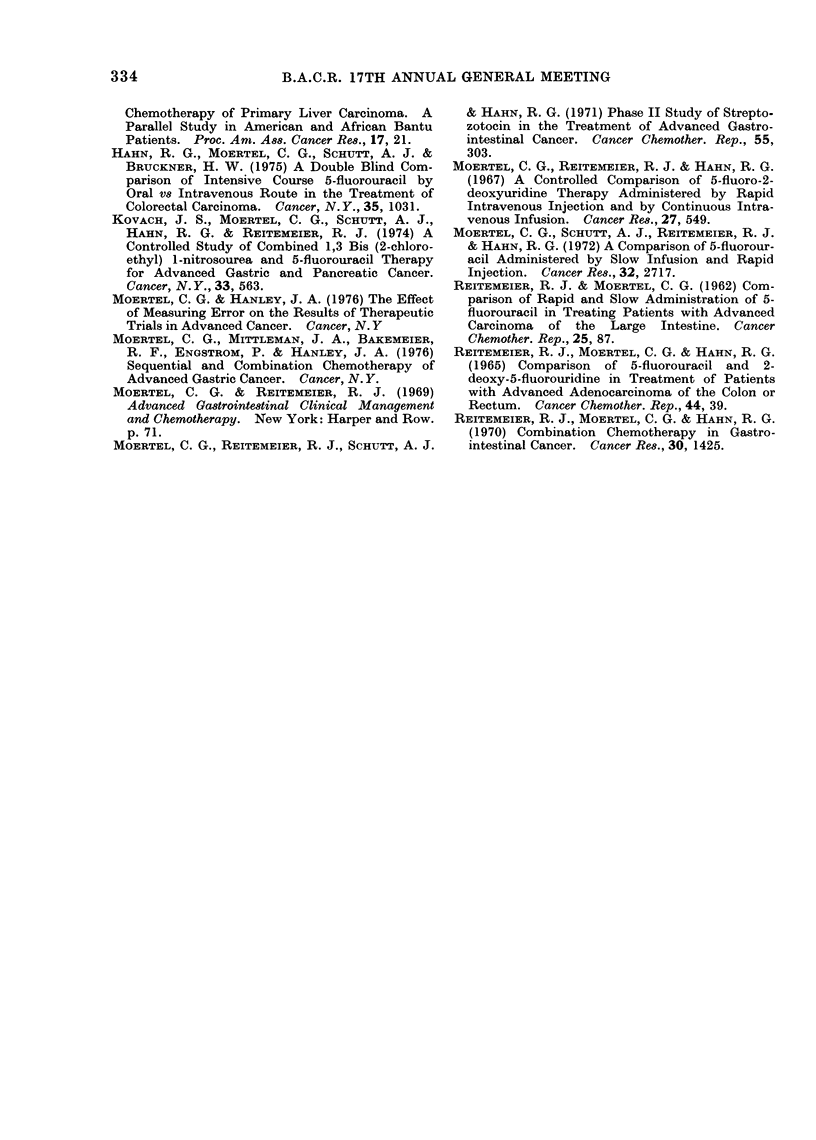# Part II: Walter Hubert Lecture Chemotherapy of Upper Gastrointestinal Carcinoma

**Published:** 1976-09

**Authors:** C. G. Moertel


### PART II: WALTER HUBERT LECTURE

### CHEMOTHERAPY OF UPPER GASTROINTESTINAL CARCINOMA

C. G. MOERTEL, *Mayo Clinic and Mayo Foundation, Rochester, Minnesota, U.S.A.*

In the Western world, carcinoma of the pancreas has waxed as gastric cancer has waned, but both remain as major causes of cancer mortality. Carcinoma of the stomach dominates the cancer incidence of Asia and the Soviet Union. Cancers of the liver and oesophagus ravage the continent of Africa in almost epidemic proportions. In spite of the great human need which this problem presents, there are few areas where the inadequacies of modern medicine are so apparent as in the management of upper gastrointestinal cancer. With all the sophistication of our diagnostic approaches, and with the ingenuity and heroism of the surgical attack, end-result statistics show that over 95% of patients found to have cancer of the oesophagus, liver, biliary tract, or pancreas will die of their disease, as will almost 90% of patients with gastric cancer. These grim statistics have changed little if at all over the past 30 years. Until methods can be evolved for either prevention or for practical detection of upper gastrointestinal carcinoma in the asymptomatic state, we must deal with the problem as the patient presents it to us, and clearly this is not at a stage amenable to surgical cure. There would not seem to be a realistic hope that advances in surgical technique *per se* can subsequently change these statistics. The results of surgery essentially plateaued a quarter of a century ago. Any progress, it would seem, must be derived from nonsurgical approaches. Among these, radiation therapy is hampered by its regional restriction and by lack of specificity. Immunotherapy has, as yet, no track record and may be largely an illusion. In spite of all its limitations, the most tangible hope in the foreseeable future would seem to rest with chemotherapy.

Chemotherapy of gastrointestinal carcinoma had its beginnings with the development in clinical application of 5-fluorouracil (5-FU) some 18 years ago. Early reports of the therapeutic effectiveness of this and other agents presented the reader with such a confusing labyrinth of claims and counterclaims that an aura of witchcraft hovered over those engaged in this field. New drugs or methods were extravagantly praised by one investigating group only to be damned by another. Objective response rates with 5-FU therapy of large bowel cancer, for example, were reported over the astounding range of 8 to 85%, even though all investigators were treating the same neoplasm with the same drug by the same dosage schedule (Moertel and Reitemeier, 1969). After these initial reports, numerous new wrinkles for administering 5-FU, or its blood-brother 5-fluoro-2′-deoxyuridine (FUdR), were devised and would predictably be advocated by the devisers as having improved therapeutic effectiveness. Such claims, however, could be regarded as little more than testimonials, since they were not validated by concurrent control groups. The reader was left with the impossible task of judging whether the reporting investigator at that point in time was an 8 percenter or an 85 percenter.

Over the past decade, the mediaeval methodology of our earlier chemotherapy trials in gastrointestinal cancer has slowly, and sometimes painfully, given way to more meaningful and reproducible scientific approaches. Although some still cling to the delusion that they can devise historical controls for retrospective comparisons, it has now become the more accepted practice for comparative clinical trials to be prospective and randomized in design. Considerable progress has also been made in defining, standardizing, and communicating the results of clinical trials. A number of factors have been elucidated which contributed to the striking variations in response rate reported in the earlier literature. For almost all areas of therapeutics it is evident that far-advanced disease, producing severe physiologic alteration, will be far less likely to respond to either surgical or medical treatment than an earlier and less devastating stage of the disease. This difference, it would seem, should be seen in even bolder relief for patients subjected to stressful cytotoxic drugs with a narrow therapeutic ratio. In Table I the results of chemotherapy of gastrointestinal cancer are related to the degree of disability of the patient at the time treatment

TABLE I.—*Relationship of Performance Status to Chemotherapy Response of the Gastrointestinal Cancer Patient*

A. Gastric cancer

| Performance status* | Patients | Objective response (%) |
|---|---|---|
| 0–1 | 66 | 39 |
| 2 | 47 | 26 |
| 3 | 21 | 4 |

B. Colorectal cancer

| Performance status* | Patients | Objective response (%) |
|---|---|---|
| 0 | 97 | 31 |
| 1 | 145 | 17 |
| 2 | 103 | 7 |
| 3 | 15 | 7 |

* ECOG Scale: 0, fully active, to 4, totally disabled.

was initiated. Disability is scored by the criteria of the Eastern Cooperative Oncology Group. A score of 0 is fully active, a score of 4, totally disabled. With a gastric carcinoma patient who is still able to work full or part-time (score 0 or 1), we get a most respectable response rate of 39%. For the patient who is up and about more than half the day but is unable to work (score 2), the response rate is almost cut in half. For the patient who is ambulatory and able to come to the office but is in a bed or chair most of the day, the response rate plummets to 4%. Precisely the same relationship is seen in the much larger group of patients with colorectal cancer. The fully ambulatory patient achieves a 31% response rate, the substantially disabled patient a 7% response rate. We do not treat totally disabled patients, but if we did, it could be surmised that we would see essentially no response at all. It can, therefore, be anticipated that an investigating group which treats largely ambulatory outpatients may achieve quite favourable responses to a given therapeutic regimen. On the other hand an investigating group who will either by their philosophy or by the nature of their practice treat largely disabled patients may report a very unfavourable therapeutic experience with precisely the same regimen.

The criteria chosen by an investigating group to declare an objective response can also have great influence on reported results. Unfortunately, in chemotherapy of gastrointestinal cancer, one only rarely sees the dramatic and complete tumour remissions that occur in lymphomas or breast cancer. In the main, we must deal with partial regressions of vaguely defined lumps and livers. A great deal of faith must be placed in the objectivity of the investigator, and even then it must be realized that he is fully subject to the human frailty of committing error. We have recently completed a study of reproducibility of measurements of lumps (Moertel and Hanley, in press). In this study the investigators measured simulated tumour masses in pairs of the same size but under circumstances where the investigators did not know they were the same. We observed striking differences in measured sizes, to the degree that an objective response would frequently be recorded for a tumour mass that had not changed at all. The salient facts of this study, which involved a number of investigators performing several hundred measurements, were that, if one used as a criterion for response a 50% reduction in the product of perpendicular diameters, an 8% response rate would be obtained due to measuring error alone (in essence, the response to a placebo). If one used just a 25% reduction criterion, as has been the practice in some studies, the placebo response rate soared to 23%. Whenever therapeutic results are reported, it is essential to scrutinize precisely what criteria were employed. It has now become a common practice to employ a 50% reduction in the product of longest perpendicular diameters as the criterion for declaring an objective response. With such a criterion, one must anticipate a 5 to 10% response due to error. These erroneous responses can be reduced by more frequent measurements, of tumour masses, or by using a longer interval between measurements, and thus taking advantage of the natural growth rate of the neoplasm. Certainly, any responses based on a 25% reduction criterion must be considered as highly suspect.

Another factor that can have a major influence on reported chemotherapy results is the method of data analysis. There are any number of ways of subjecting results to statistical massage; and if you are skilful at playing this game, it is remarkably easy to turn a sow's ear into a silk purse. One of the most frequent ploys is the unevaluable patient gambit. The technique is to take patients who start on treatment but who become too sick to continue or who die soon after and discard these from your analysis as "unevaluable". In some reports one-third

TABLE II.—*Comparative Survival Times Among Specific Types of Gastrointestinal Adenocarcinomas Measured From Time of Proof of Incurable Cancer*

| Primary tumour | No. of patients | Survival: Median (months) | Survival: Range |
|---|---|---|---|
| Hepatoma (cirrhosis) | 15 | 2·0 | 1 week to 40 months |
| Gall bladder | 114 | 2·5 | 1 week to 23 months |
| Pancreas | 145 | 3·5 | 4 weeks to 10 years |
| Stomach | 307 | 4·0 | 4 weeks to 12 years |
| Duodenum | 15 | 5·0 | 3 weeks to 13 months |
| Bile ducts | 59 | 5·0 | 2 weeks to 43 months |
| Ampulla of Vater | 22 | 6·0 | 6 weeks to 39 months |
| Jejunum and ileum | 28 | 9·0 | 5 weeks to 34 months |
| Carcinoid (small bowel) | 35 | 30·0 | 8 weeks to 23 years |
| Islet cell carcinoma | 6 | 57·0 | 5 months to 13 years |

to one-half of the original patients are swept under the table in this manner, and this, of course, greatly inflates the response rate. Such a legerdemain, however, would seem to be a rather questionable practice. If a patient becomes too sick to continue treatment, or if he dies, he would seem as evaluable as he can get. Indeed, it is quite possible that adverse effects of treatment could have contributed to his rapid deterioration. If a patient is selected for treatment and initiated on treatment, he deserves a place in the denominator.

### *The natural history of untreated upper gastrointestinal carcinoma*

Before discussing treatment, we should first have some concept of the natural history of the disease we are treating, at least in terms of survival. This provides us with an essential background against which we can view our therapeutic accomplishments. Table II shows the longevity of untreated patients with upper gastrointestinal carcinoma, measured from the time of proof of unresectable disease. There is a striking range in longevity among the specific tumour types, ranging from the virulent hepatomas, complicating cirrhosis, and carcinoma of the gallbladder, to the remarkably indolent carcinoid tumours and islet-cell carcinomas. It can also be seen that there is a striking range in longevity within each tumour type. It is worth emphasis that some of these patients can have exceedingly long survivals with no treatment whatsoever. In Table III the more specific characteristics of the patient and his disease that have a significant influence on prognosis are considered. Here we have employed our more frequent upper gastrointestinal carcinomas, cancer of the stomach and cancer of the pancreas. These determinants are particularly important in providing appropriate stratification for randomized trials. We found that age and sex of the patient had little influence on longevity except that the very elderly, 75 years or older, tended to have shorter survivals. As might be expected, the patients with only regional disease lived longer than those with distant abdominal spread, who lived longer than those with hepatic metastasis, who lived longer than those with extra-abdominal metastasis. Location of the primary lesion in the stomach had no influence, but patients with carcinoma of the head of the pancreas

TABLE III.—*Clinical Characteristics and Survival in Patients with Unresectable Gastric and Pancreatic Carcinoma*

| Characteristic | Median survival (months): Gastric | Median survival (months): Pancreatic |
|---|---|---|
| Extent of disease | | |
| Regional | 6·0 | 5·0 |
| Abdominal | 3·0 | 4·0 |
| Hepatic | 2·5 | 2·5 |
| Extra-abdominal | 2·0 | 2·0 |
| Location of primary (pancreas) | | |
| Head | — | 4·0 |
| Body | — | 3·0 |
| Tail | — | 2·5 |
| Grade of anaplasia (Broder's) | | |
| 1 and 2 | 7·0 | 5·5 |
| 3 and 4 | 4·0 | 3·0 |
| Interval, resection to recurrence | | |
| 1 to 5 months | 2·0 | — |
| 6 to 11 months | 3·0 | — |
| 12 to 23 months | 6·0 | — |
| 24 months and over | 6·5 | — |
| Palliative surgery | | |
| None | 3·5 | 2·0 |
| Bypass | 4·5 | 4·5 |
| Resection | 6·0 | — |

lived longer than those with primaries in the body or tail—perhaps because the tumour in the head is heralded at an earlier stage by the development of obstructive jaundice. Also, as might be expected, patients with highly anaplastic neoplasms had a much more rapid course than those with a well-differentiated microscopic morphology. Again, perhaps proving the obvious, malignant disease that is indolent in its earlier phases tends to be indolent throughout its course. In gastric cancer, this can be demonstrated by the interval from resection of the primary with curative intent to proof of metastases; in pancreatic carcinoma, by a less precise measure, the duration of symptoms. If the surgeon performs a palliative procedure, such as a bypass or resection, the patient does live longer. This may be artifactual, however, since in the patient with very advanced disease, the surgeon may consider a palliative attempt either injudicious or technically impossible.

We have also found that assessments of immune function have prognostic significance. Elevated immunoglobulins indicate a very short survival. The patient with lesser numbers of T cells or impaired T-cell function also lives a shorter period of time. Such patients with impaired laboratory parameters of immune function, however, are also patients with advanced stages of disease associated with impaired nutrition and impaired performance status. We are not convinced at this time that these elaborate and expensive immunologic assays tell us more than can be discerned by already apparent clinical determinations.

*Treatment of gastrointestinal cancer with 5-fluorouracil*

5-fluorouracil (5-FU) was the first chemotherapeutic agent to prove capable of inducing tumour regressions of gastrointestinal adenocarcinoma in the hands of all investigators reporting its use. Although the true therapeutic value of this agent may be questioned, it has nevertheless served as a stimulus and catalyst for clinical research in the treatment of this most common group of neoplasms.

In an attempt to improve the therapeutic ratio of 5-FU, this agent has been delivered through every natural or artificial orifice into almost every available human lumen by every conceivable dosage schedule. Predictably, each new approach has been lauded by the initiator as more effective, less toxic, and eminently suitable for office practice. Equally predictably, none of these claims have stood the test of a controlled clinical trial. Noteworthy conclusions of these controlled trials have been the following: (1) the nucleoside of 5-FU, 5 fluoro-2′-deoxyuridine (FUdR), does not have any significant clinical advantage over 5-FU (Reitemeyer, Moertel and Hahn, 1965); (2) slow infusion of these fluorinated pyrimidines adds expense and nuisance to the treatment procedure but not improved therapeutic efficacy (Reitemeyer and Moertel, 1962; Moertel, Reitemeyer and Hahn, 1967; Moertel *et al.*, 1972); (3) the oral route of administration of 5-FU is associated with erratic drug absorption and significantly inferior therapeutic effect, regardless of administration schedule (Hahn *et al.*, 1975; Bateman *et al.*, 1975; Ansfield, 1975) and (4) weekly administration of 5-FU is significantly inferior to the loading course method (Ansfield, 1975).

Table IV would seem to put the fluorinated pyrimidine question into proper prospective. Here we have related the objective response rates to 5-FU or FUdR, regardless of how they were given, to degree of toxicity as measured by nadir of leucopenia. It is evident that if no toxicity at all is experienced, results are inferior to those when patients are treated to mild or moderate toxicity. On the other hand, treating to severe or nearly lethal toxicity does not improve, but rather seems to detract from, therapeutic effect. It must be concluded that 5-FU or FUdR are not cancer-specific drugs. Any significant effect on the neoplastic tissue must be achieved at the price

TABLE IV.—*Toxicity and Objective Response to Fluorinated Pyrimidine Therapy*

A. Gastric cancer (110 patients)

| Nadir of leucopenia | Objective response (%) | |
|---|---|---|
| $4000/mm^3$ | 26 | $P < 0{\cdot}05$ |
| 4000 to $1500/mm^3$ | 41 | |
| $1500/mm^3$ | 13 | |

B. Colorectal cancer (381 patients)

| Nadir of leucopenia | Objective response (%) | |
|---|---|---|
| $4000/mm^3$ | 9 | $P < 0{\cdot}01$ |
| 4000 to $1500/mm^3$ | 23 | |
| $1500/mm^3$ | 15 | |

of some toxic effect to normal tissues. A severe cytotoxic effect, however, seems to be more detrimental to host than to tumour.

### CHEMOTHERAPY OF GASTRIC AND PANCREATIC CARCINOMA

*Single agent therapy*

To this date, our primary task in chemotherapy of pancreatic and gastric carcinoma has been a search for single agents that will show some evidence of significant activity when used alone and, hopefully, will form the basis for a later evolution of combination drug therapy regimens. In this quest, we have followed largely an empirical road.

Table V shows our single-agent experiences with advanced pancreatic carcinoma. These have been singularly discouraging.

TABLE V.—*Chemotherapy of Advanced Pancreatic Carcinoma: Single Agents*

| Regimen | Patients | Objective response | Median duration of response (months) |
|---|---|---|---|
| 5-FU | 39 | 6 (15%) | 2·5 |
| BCNU | 18 | — | |
| Methyl CCNU | 15 | 2 (13%) | 8·5 |
| Actinomycin D | 13 | — | |
| MTX (i.v.) | 13 | 1 (8%) | |
| Adriamycin | 11 | 1 (9%) | |
| Fluorometholone | 7 | — | |
| MTX (Oral) | 6 | — | |
| Misc. (< 3 each) | 12 | 1* | |

* Single patient treated with FUdR.

5-FU produces only a minimum response rate and the responses are exceedingly transitory. Bischloroethyl nitrosourea (BCNU) and methyl chloroethyl cyclohexyl nitrosourea (methyl CCNU) have been equally discouraging. None of the other single drugs at which we or others have looked show anything more than an occasional spurious response.

In quite remarkable contrast are our results with single-agent therapy of gastric carcinoma (Table VI). Here we have much more respectable response rates and these with agents of a variety of specific activities. 5-FU produces a very acceptable rate and duration of regression. Surprisingly we have achieved an excellent response rate with adriamycin, a drug that is essentially worthless for colorectal cancer. Even with this brighter picture in gastric carcinoma, however, it would seem quite clear that single-drug therapy has not provided substantive benefit in any type of gastrointestinal cancer. Much of our more recent efforts have, therefore, been directed towards combination drug therapy. As a rule of thumb, we feel that for drugs to be effective in combination, they must show some evidence of activity when used alone. Combining ineffective drugs has inevitably led to ineffective combinations. Also, we have felt that one must be able to use the constituent drugs together with a less than proportionate summation of their toxic effects. Our first study in this regard was an evaluation of combinations involving 5-FU, Mitomycin C, and BCNU. In pilot studies, we found that when Mitomycin C and BCNU were used in double combination, you could use only one-half of a full dose of each. Mitomycin C and 5-FU looked a bit better, permitting two-thirds of a full dose of each. 5-FU and BCNU look particularly attractive since 75% of the full dose could be combined with no more toxicity than using either of the drugs alone. With this background, we then initiated a controlled evaluation, randomizing 215 patients with gastrointestinal cancer to treatment with each of the single drugs, with each of the three possible double drug combinations, and with the triple drug combination (Reitemeier, Moertel and Hahn, 1970). Regrettably, none of the combinations performed any better than 5-FU alone and most of them were worse. We were particularly disappointed with combined 5-FU and BCNU which had demonstrated only a minor and insignificant increase in duration of response. In this study, as was common practice in that era, we put all the specific types of gastrointestinal cancers into the same pot in the

TABLE VI.—*Chemotherapy of Advanced Gastric Carcinoma: Single Agents*

| Regimen | Patients | Objective response | Median duration of response (months) |
|---|---|---|---|
| 5-FU | 72 | 19 (26%) | 4·5 |
| BCNU | 33 | 6 (18%) | 4·0 |
| Adriamycin | 29 | 11 (38%) | 5·0 |
| Methyl CCNU | 15 | 2 (13%) | 4·0 |
| Fluorometholone | 14 | 1 (7%) | |
| CCNU | 11 | — | |
| Mitomycin C | 11 | 3 (27%) | 2·7 |
| Camptothecin | 7 | — | |
| Hydroxyurea | 6 | 1 | |
| Streptonigrin | 5 | 1 | |

belief that they would all respond the same. In recent years, it has become evident that this is an erroneous assumption. All gastrointestinal cancers are not alike. When we looked at our responses of specific cancers to combined 5-FU and BCNU, we found that the response rate for colorectal cancer was abominable and this negative observation has subsequently been confirmed by Lokich and associates. On the other hand, the response rate in a very small group of gastric and pancreatic carcinomas looked remarkably good. With this encouragement we expanded our controlled study to a much larger group of 167 gastric and pancreatic cancer patients randomized to 5-FU alone, BCNU alone, and the 5-FU/BCNU combination (Table VII)

TABLE VII.—*A Controlled Evaluation of Combined 5-FU and BCNU Therapy for Advanced Gastric and Pancreatic Carcinoma*

| Primary site | Regimen | Patients | Objective response (%) |
|---|---|---|---|
| Stomach | 5-FU | 28 | 29 |
| | BCNU | 23 | 17 |
| | 5-FU + BCNU | 34 | 41 |
| Pancreas | 5-FU | 31 | 16 |
| | BCNU | 21 | 0 |
| | 5-FU + BCNU | 30 | 33 |

(Kovach *et al.*, 1974). In gastric cancer, 41% of patients responded to the combination compared to 29% and 17% with the single drugs. In pancreatic cancer it was 33% *vs* 16% and 0%. If we take the liberty of grouping these two sets of data and calling them all upper gastrointestinal carcinoma, then the superiority of the 5-FU/BCNU combination approaches statistical significance. But to be really clinically significant, objective response must translate into a favourable change in the natural history of the disease. The only objective means of measuring this is in terms of patient survival. When we analysed our survival curves for carcinoma of the pancreas, we found that all overlapped and they were essentially the same as that of clinically matched untreated patients. For gastric cancer, however, the picture was a bit brighter. BCNU alone clearly added nothing, but both 5-FU and the combination were associated with improved early suvival. This quickly faded with 5-FU, but it continued with the combination. At one and one-half years 27% of these patients with far advanced gastric cancer treated with 5-FU plus BCNU were still living compared to only 7% with either single drug or with no treatment at all.

With this glimmer of success, we then turned our attention to the newer nitrosourea analogues, particularly to methyl CCNU. This drug has the advantage of an oral route of administration whereas BCNU has to be given i.v. Also, methyl CCNU seems to have some therapeutic advantage in animal models. In the Eastern Cooperative Oncology Group, therefore, we initiated a study in gastric cancer comparing methyl CCNU alone with the combination of 5-FU and methyl CCNU (Moertal *et al.*, in press). In this study we threw in the wrinkle of cyclophosphamide induction, and that was a total failure (Table VIII). The combination of

TABLE VIII.—*Therapy of Advanced Gastric Cancer: Methyl CCNU Alone* vs *5-FU Methyl CCNU; with An Evaluation of Cyclophosphamide (CTX) Induction*

| Regimen | Patients | Objective response (%) |
|---|---|---|
| CTX → methyl CCNU | 30 | 7 |
| Methyl CCNU alone | 37 | 8 |
| CTX→5-FU+methyl CCNU | 30 | 20 |
| 5-FU+methyl CCNU | 30 | 40* |

* 5-FU + methyl CCNU superior to all other regimens, $P < 0 \cdot 05$.

5-FU and methyl CCNU, however, garnered a 40% objective response, significantly superior to that achieved with methyl CCNU alone. In addition, survival of patients treated with the combination was also significantly superior to that for patients treated with the single drug. Further confirmation of the effectiveness of this combination has come from studies by the Southwest Oncology Group (Baker *et al.*, 1975) (Table IX). This study involved a spectrum of upper gastrointestinal carcinomas and compared the

TABLE IX.—*A Comparison of 5-FU + Methyl CCNU* vs *5-FU Alone in Upper Gastrointestinal Carcinoma (Southwest Oncology Group), 5-FU given by weekly injection*

| Regimen | Patients | Objective response (%) |
|---|---|---|
| 5-FU alone | 22 | 9 |
| 5-FU + Methyl CCNU | 53 | 25 |

5-FU/methyl CCNU combination with 5-FU used alone. In this study the weekly method for 5-FU was employed; and perhaps because of this, overall response rates were scaled down. But again, there was a substantial advantage for the combination in comparison to 5-FU alone. With 3 successive studies showing improvement in response rate with the combination compared to single drug treatment, and with 2 successive studies demonstrating significant improvement in survival, I believe we are now justified in concluding that combined 5-FU and nitrosourea therapy is a significant advance in the management of gastric cancer. The question remains to be answered whether we can do more with this combination than just shrink lumps and delay death. Combined 5-FU and methyl CCNU as an adjuvant to potentially curative gastric cancer surgery is now undergoing clinical trial in the Gastrointestinal Tumour Study Group, the Eastern Cooperative Oncology Group, and the Veterans Administration Surgical Adjuvant Group.

*Combination chemotherapy of pancreatic carcinoma*

Table X shows overall experience with combination chemotherapy of advanced pancreatic carcinoma. The best results we have obtained to date have been with the 5-FU/BCNU combination, and this, as mentioned above, did not produce any improvement in patient longevity. The other combinations employed have added little if anything to the meagre accomplishments of 5-FU used alone. The primary task in pancreatic carcinoma remains a search for significant therapeutic activity.

TABLE X.—*Chemotherapy of Advanced Pancreatic Carcinoma with Drug Combinations*

| Regimen | Patients | Objective response | Median duration of response (months) |
|---|---|---|---|
| 5-FU + BCNU | 21 | 7 (33%) | 5·5 |
| 5-FU + streptozotocin | 11 | 2 (18%) | 6·0 |
| 5-FU + cyclophosphamide | 10 | 1 (10%) | |
| 5-FU + fluorometholone | 7 | 1 (14%) | |
| 5-FU + Mitomycin C | 3 | — | |

*Chemotherapy of oesophageal carcinoma*

Squamous cell carcinoma is not an uncommon malignant disease and surgical results have been characterized by an operative mortality rate that exceeds the cure rate. It is surprising that chemotherapy experience with this disease has been so meagre. Bleomycin has perhaps had the largest overall clinical application. The reported response rates with this drug, however, are far from impressive. Among 14 consecutive patients we treated with bleomycin as a single agent, we did not observe any evidence of therapeutic activity. Our only other trial has been with the nitrosourea, CCNU. Here we observed 3 responses among 19 patients treated and these persisted for periods of only 2, 3 and 9 months. Certainly this is nothing approaching a therapeutic accomplishment. Our present effort in oesophageal carcinoma must be in Phase II evaluations of single drugs, with the hope that we can unearth at least a few agents with some degree of activity that can form the constituents of rational drug combinations.

*Chemotherapy of hepatocellular carcinoma* (Table XI)

We have had occasional brief responses with systemic 5-FU, and others have noticed occasional brief responses with 5-FU or FUdR by hepatic artery infusion. Studies at the University of Wisconsin (Davis, Ramirez and Ansfield, 1974), however, have shown that the expensive and cumbersome intra-arterial approaches really contribute nothing significant to patient survival. Of special interest to us has been the quite impressive response rate we have achieved with combined 5-FU and BCNU therapy.

TABLE XI.—*Chemotherapy of Hepatoma*

| Regimen | Patients | Objective response | Duration of response (months) |
|---|---|---|---|
| 5-FU | 8 | 3 (37%) | 2, 2, 9 |
| Adriamycin | 7 | 2 | 2, 3½ |
| Mitomycin C | 4 | 1 | 5 |
| BCNU | 3 | — | |
| Fluorometholone | 3 | — | |
| Methotrexate | 2 | — | |
| Emetine | 2 | — | |
| 5-FU + BCNU | 19 | 7 (37%) | 2, 2, 4, 4, 25+, 44+, 75 |

Thirty-seven per cent of our patients have shown tumour regression and 3 of these were of very long duration. In a recently completed joint African and American study of hepatoma therapy (Falkson, Moertel and Lavin, 1976), adriamycin as well as the combinations of 5-FU plus each of the 2 nitrosoureas, methyl CCNU and streptozotocin, all produced a significant improvement in survival of hepatoma patients when compared to treatment with 5-FU alone. We are now most interested in pursuing the 3 drug combinations of 5-FU, methyl CCNU and adriamycin.

*Chemotherapy of the carcinoid tumour and the malignant carcinoid syndrome*

The malignant carcinoid syndrome provides a dramatic event in oncologic practice. We must, however, restrain our zeal to expose these patients to the hazards of cytotoxic drugs at an early stage of the disease. This tumour is exceedingly indolent and patients can frequently have several years of productive life before the disease causes them any real disability. Although the carcinoid syndrome may be fascinating to the physician, its early stages seldom cause the patient more problems than an occasional blush and mild diarrhoea which can be controlled with standard symptomatic measures. All the patients listed Table XII, therefore, had far advanced disease and yet 5-FU alone produced a reasonable rate of response. A few years ago we reported therapeutic activity with streptozotocin (Moertel *et al.*, 1971), and this has subsequently been confirmed by others. Responses to this agent, however, are usually incomplete and short-lasting. Since streptozotocin has hardly any haematological toxicity, it can be combined with other cytotoxic drugs in essentially full doses of each agent. Our rate of response with a cyclophosphamide/streptozotocin combination was very disappointing. It was, however, exceedingly favourable for the 5-FU/streptozotocin combination. Both of these combinations are currently undergoing more extensive evaluation in an Eastern Cooperative Oncology Group Study.

TABLE XII.—*Chemotherapy of Carcinoid Tumour*

| Regimen | Patients | Objective response |
|---|---|---|
| 5-FU | 20 | 6 (30%) |
| Actinomycin D | 9 | 1 (11%) |
| Streptozotocin | 6 | 3 (50%) |
| Phenylalanine mustard | 6 | — |
| Fluorometholone | 5 | — |
| Adriamycin | 4 | 1 (25%) |
| Mitomycin C | 3 | — |
| Cyclophosphamide (CTX) | 2 | — |
| 5-FU + streptozotocin | 13 | 6 (46%) |
| CTX + streptozotocin | 11 | 1 (9%) |
| 5-FU + BCNU | 2 | — |
| BCNU + streptozotocin | 1 | 1 |

Great caution must be exercised in offering effective chemotherapy to the patient with the malignant carcinoid syndrome, since this may trigger off a severe and life-threatening carcinoid crisis. We have not found any serotonin antagonist to combat this complication effectively. In treatment of the carcinoid syndrome, it is therefore our policy to initiate therapy with one-half the projected therapeutic dose in patients who have florid manifestations of the syndrome or who have urine 5-hydroxyindolacetic levels greater than 150 mg/24 h.

*Chemotherapy of islet-cell carcinoma*

Much akin to the carcinoid is the islet-cell carcinoma, a tumour with a variety of functional capacities. As in the carcinoid, we have seen an occasional response with 5-FU (Table XIII). Streptozotocin, however, has written a new chapter in the treatment of these neoplasms. Among a collected series of 56 patients with hormonally active islet-cell carcinomas reported by Broder and Carter (1973), functional improvement was observed in 64% and objective tumour regression was seen in 37%. We have observed 3 objective responses among 6 patients, one of which was most

TABLE XIII.—*Chemotherapy of Islet-cell Carcinoma*

| Regimen | Patients | Objective response | Duration of response (months) |
|---|---|---|---|
| Streptozotocin | 8 | 3 | 7, 9½, 16½+ |
| Methyl CCNU | 6 | 2 | 2, 14 |
| Tubercidin | 5 | 2 | 2, 3 |
| Adriamycin | 4 | 1 | 9 |
| 5-FU | 3 | 1 | 8 |
| Streptozotocin + 5-FU | 10 | 7 | 3, 3½, 4+, 8½, 12+, 53+, 55+ |

dramatic, in a patient near death with a fulminating pancreatic cholera syndrome, showing a high rate response to the 5-FU/ streptozotocin combination. Some of these responses have also been of a very striking nature and of very long duration. This combination is currently being compared in a controlled study with streptozotocin used alone.

### CONCLUSIONS

Until the very recent past, chemotherapy of upper gastrointestinal cancer was largely an academic endeavour with no evidence of substantive contribution to the overall population of patients treated, whether measured in terms of symptomatic palliation or improved survival time. Now, however, it is possible to recognize a few definite steps of progress. Refinements of methodology have allowed data to be communicated in a meaningful way. In comparative trials, the randomized prospective design now permits believable differences to become apparent without the cloud of subjectivity and artefacts that obscures the testimonial-type reports or the study based only on historical controls. Although 5-FU probably remains the most active single drug in upper gastrointestinal cancer, definite antineoplastic effect has been demonstrated for a number of other agents. It does, however, seem obvious that no single drug therapy has been of significant value for the upper gastrointestinal cancer patient, whatever the stage of the disease at which it is administered. Following the path that has led to success in leukaemia, lymphoma and breast cancer, increasing emphasis is now placed on combination chemotherapy regimens for upper gastro-intestinal cancer. Combined 5-FU and nitrosourea therapy has increased response rate for carcinomas of the stomach, pancreas, and liver to the 30 to 40% range. In gastric carcinoma and in hepatoma this has also been associated with improved patient survival. Combined 5-FU and streptozotocin has produced even higher response rates in the carcinoid tumour and islet-cell carcinoma.

Even though these results may seem exciting, they are certainly far from our ultimate objectives. Responses in advanced disease are still transient and all the patients still die of their disease. None of our approaches for advanced and metastatic upper gastrointestinal cancer are of sufficient value to justify offering as standard or routine treatment. We must continue to regard all patients at this stage of their disease as subjects for clinical research.

It is obvious that the only hope for increasing cure rates for upper gastrointestinal cancer in the foreseeable future lies in the application of chemotherapy, or perhaps immunotherapy, to the patient who has been brought to the point of minimal tumour burden by surgical resection. Surgical adjuvant chemotherapy attempts in the past have been notable only for their failures. The chemotherapy applied under these circumstances, however, has had very little evidence of therapeutic activity, *e.g.* thiotepa, 5-FU, FUdR and Mitomycin C. With the significantly greater activity now demonstrated by our combination regimens, it would seem appropriate that new efforts at surgical adjuvant therapy be undertaken.

In treating the patient with upper gastrointestinal cancer, it is evident that neither surgeon, radiotherapist, nor oncologist have accomplished anything of real value by working alone or in sequence, nor is there any realistic hope that such solo performances will produce any substantive future accomplishment. If, however, we can offer our patients the best of all concerned disciplines, working together in carefully designed protocols, then I feel we can confidently anticipate significant improvements in the results of treatment for tomorrow's upper gastrointestinal cancer patient.

### REFERENCES

ANSFIELD, F. J. (1975) A Randomized Phase III Study of Four Dosage Regimens of 5-fluorouracil. A Preliminary Report. *Proc. Am. Ass. Cancer Res.*, **16**, 224.

BAKER, L. H., MATTER, R., TALLEY, R. & VAITKEVICIUS, V. (1975) 5-FU vs 5-FU and MeCCNU in Gastrointestinal Cancer. *Proc. Am. Ass. Cancer Res.*, **16**, 229.

BATEMAN, J., IRWIN, L., PUGH, R., CASSIDY, F. & WEINER, J. (1975) Comparison of Intravenous and Oral Administration of 5-fluorouracil for Colorectal Carcinoma. *Proc. Am. Ass. Cancer Res.*, **16**, 242.

BRODER, L. F. & CARTER, S. K. (1973) Pancreatic Islet Cell Carcinoma. II. Results of Therapy with Streptozotocin. 52 Patients. *Ann. int. Med.*, **79**, 108.

DAVIS, H. L., JR, RAMIREZ, G. & ANSFIELD, F. J. (1974) Adenocarcinoma of Stomach, Pancreas, Liver and Biliary Tracts. *Cancer, N.Y.*, **33**, 229.

FALKSON, G., MOERTEL, C. G., LAVIN, P. T. (1976)

Chemotherapy of Primary Liver Carcinoma. A Parallel Study in American and African Bantu Patients. *Proc. Am. Ass. Cancer Res.*, **17**, 21.

HAHN, R. G., MOERTEL, C. G., SCHUTT, A. J. & BRUCKNER, H. W. (1975) A Double Blind Comparison of Intensive Course 5-fluorouracil by Oral *vs* Intravenous Route in the Treatment of Colorectal Carcinoma. *Cancer, N.Y.*, **35**, 1031.

KOVACH, J. S., MOERTEL, C. G., SCHUTT, A. J., HAHN, R. G. & REITEMEIER, R. J. (1974) A Controlled Study of Combined 1,3 Bis (2-chloroethyl) 1-nitrosourea and 5-fluorouracil Therapy for Advanced Gastric and Pancreatic Cancer. *Cancer, N.Y.*, **33**, 563.

MOERTEL, C. G. & HANLEY, J. A. (1976) The Effect of Measuring Error on the Results of Therapeutic Trials in Advanced Cancer. *Cancer, N.Y*

MOERTEL, C. G., MITTLEMAN, J. A., BAKEMEIER, R. F., ENGSTROM, P. & HANLEY, J. A. (1976) Sequential and Combination Chemotherapy of Advanced Gastric Cancer. *Cancer, N.Y.*

MOERTEL, C. G. & REITEMEIER, R. J. (1969) *Advanced Gastrointestinal Clinical Management and Chemotherapy*. New York: Harper and Row. p. 71.

MOERTEL, C. G., REITEMEIER, R. J., SCHUTT, A. J. & HAHN, R. G. (1971) Phase II Study of Streptozotocin in the Treatment of Advanced Gastrointestinal Cancer. *Cancer Chemother. Rep.*, **55**, 303.

MOERTEL, C. G., REITEMEIER, R. J. & HAHN, R. G. (1967) A Controlled Comparison of 5-fluoro-2-deoxyuridine Therapy Administered by Rapid Intravenous Injection and by Continuous Intravenous Infusion. *Cancer Res.*, **27**, 549.

MOERTEL, C. G., SCHUTT, A. J., REITEMEIER, R. J. & HAHN, R. G. (1972) A Comparison of 5-fluorouracil Administered by Slow Infusion and Rapid Injection. *Cancer Res.*, **32**, 2717.

REITEMEIER, R. J. & MOERTEL, C. G. (1962) Comparison of Rapid and Slow Administration of 5-fluorouracil in Treating Patients with Advanced Carcinoma of the Large Intestine. *Cancer Chemother. Rep.*, **25**, 87.

REITEMEIER, R. J., MOERTEL, C. G. & HAHN, R. G. (1965) Comparison of 5-fluorouracil and 2-deoxy-5-fluorouridine in Treatment of Patients with Advanced Adenocarcinoma of the Colon or Rectum. *Cancer Chemother. Rep.*, **44**, 39.

REITEMEIER, R. J., MOERTEL, C. G. & HAHN, R. G. (1970) Combination Chemotherapy in Gastrointestinal Cancer. *Cancer Res.*, **30**, 1425.